# Effect of myocardial heterogeneity on ventricular electro-mechanical responses: a computational study

**DOI:** 10.1186/s12938-019-0640-7

**Published:** 2019-03-14

**Authors:** Nida Dusturia, Seong Wook Choi, Kwang Soup Song, Ki Moo Lim

**Affiliations:** 1 Department of IT Convergence Engineering, Kumoh National Institute of Technology, 61 Daehak-ro, Gumi, Gyeongbuk 39253 Republic of Korea; 2 Department of Mechanical and Biomedical Engineering, Kangwon National University, Chuncheon, Republic of Korea; 3 Department of Medical IT Convergence Engineering, Kumoh National Institute of Technology, Gumi, Republic of Korea

**Keywords:** Cardiac arrhythmia, Endocardium, Epicardium, Heterogeneous ventricular models, Mid-myocardium

## Abstract

**Background:**

The heart wall exhibits three layers of different thicknesses: the outer epicardium, mid-myocardium, and inner endocardium. Among these layers, the mid-myocardium is typically the thickest. As indicated by preliminary studies, heart-wall layers exhibit various characteristics with regard to electrophysiology, pharmacology, and pathology. Construction of an accurate three-dimensional (3D) model of the heart is important for predicting physiological behaviors. However, the wide variability of myocardial shapes and the unclear edges between the epicardium and soft tissues are major challenges in the 3D model segmentation approach for identifying the boundaries of the epicardium, mid-myocardium, and endocardium. Therefore, this results in possible variations in the heterogeneity ratios between the epicardium, mid-myocardium, and endocardium. The objective of this study was to observe the effects of different thickness ratios of the epicardium, mid-myocardium, and endocardium on cardiac arrhythmogenesis, reentry instability, and mechanical responses during arrhythmia.

**Methods:**

We used a computational method and simulated three heterogeneous ventricular models: Model 1 had the thickest M cell layer and thinnest epicardium and endocardium. Model 2 had intermediate layer thicknesses. Model 3 exhibited the thinnest mid-myocardium and thickest epicardium and endocardium. Electrical and mechanical simulations of the three heterogeneous models were performed under normal sinus rhythm and reentry conditions.

**Results:**

Model 1 exhibited the highest probability of terminating reentrant waves, and Model 3 exhibited to experience greater cardiac arrhythmia. In the reentry simulation, at 8 s, Model 3 generated the largest number of rotors (eight), while Models 1 and 2 produced five and seven rotors, respectively. There was no significant difference in the cardiac output obtained during the sinus rhythm. Under the reentry condition, the highest cardiac output was generated by Model 1 (19 mL/s), followed by Model 2 (9 mL/s) and Model 3 (7 mL/s).

**Conclusions:**

A thicker mid-myocardium led to improvements in the pumping efficacy and contractility and reduced the probability of cardiac arrhythmia. Conversely, thinner M cell layers generated more unstable reentrant spiral waves and hindered the ventricular pumping.

## Background

The regional heterogeneity within the heart is examined with regard to the electrophysiological and mechanical properties [1]. The heart wall is anatomically divided into three layers. The outermost thin surface corresponds to the epicardium, the thin inner layer of the heart corresponds to the endocardium, and the middle heart layer corresponds to cardiac muscle tissue and is commonly known as the mid-myocardium [2, 3]. These layers are distinguished by their electrophysiological, pharmacological, and pathological properties [4]. For example, owing to differences in the morphology of the action potential (AP), ventricular epicardial cells exhibit a shorter AP duration (APD) than endocardial cells [1, 5].

Seemann et al. [4] reported that regional electrophysiological heterogeneity in a myocardium resulted in different ion-channel kinetics across the cardiac wall. This affected the phases of the cardiac AP, including the plateau, repolarization phase, and force development in the heart. Another recent study based on data from Tusscher et al. [6] indicated that the epicardium, mid-myocardium, and endocardium had different AP shapes owing to differences in the ion-channel density. The results suggested that the epicardium and endocardium had similar AP morphologies and durations, while the mid-myocardial layers had the longest APD. Myocardial infarction can also cause myocardium heterogeneity because it affects the mechanical properties and impairs the contractility of the myocardium [7, 8].

It is difficult to precisely define the topographical distribution of M cells [9], because the topography and anatomic locations of mid-myocardial populations can vary in the heart [10, 11]. For example, M cells are more prominent in the anterior left ventricular wall than in the posterior left ventricular wall [12]. Given the differences in the distribution of M cells, the possible thickness variations of the mid-myocardial layers cannot be ignored.

Construction of an accurate three-dimensional (3D) heart model is important for visualizing the anatomical structures and predicting the physiological behaviors of the heart [13–15]. The construction of a 3D cardiac model requires the identification of the boundaries of the ventricular epicardium and endocardium via segmentation approaches [15]. Furthermore, the delineation of the ventricular myocardium characteristics plays an important role in the prognosis and diagnosis of the cardiac diseases [7, 16]. The major challenges faced by researchers are related to the wide variabilities of the myocardial shapes, the unclear edges between the epicardium and soft tissues, and the quality of cardiac images obtained via computed tomography and magnetic resonance (MR) imaging [16]. Therefore, some approaches are employed to identify and segment the epicardium and endocardium [16–18]. This can result in variations of thickness-ratios of the epicardium and endocardium.

Regarding the computational model of the heart, cardiac electromechanical models have been developed and aid researchers in understanding the underlying cardiac electrophysiology and the mechanism of arrhythmogenesis [19–22]. Initial heart modeling indicated that the ventricles were formed with geometries of elliptical and cylindrical shapes [23–26]. These studies provided insights regarding the effects of the volume of the ventricular chamber, the fiber geometry, and the wall thickness on the ventricular mechanics. Moreover, Gurev et al. presented a sophisticated electromechanical model of the heart, which included MR and diffusion tensor MR (DTMR) image segmentation, the generation of electrical and mechanical finite-element meshes, and a coupling model between the mechanical compartment and the circulatory system [27].

In summary, the three layers of the heart wall exhibit different characteristics with regard to anatomy, topography, and electrophysiology. The purpose of this study was to observe the effects of different myocardial heterogeneities of human ventricles (i.e., different thickness ratios of the epicardium, mid-myocardium, and endocardium) on cardiac arrhythmogenesis, the instability of reentry, and the mechanical responses during arrhythmia. We used a 3D electromechanical model of the human heart and evaluated the electrical and mechanical responses of ventricular models.

## Methods

### Description of computational model

**Fig. 1:**
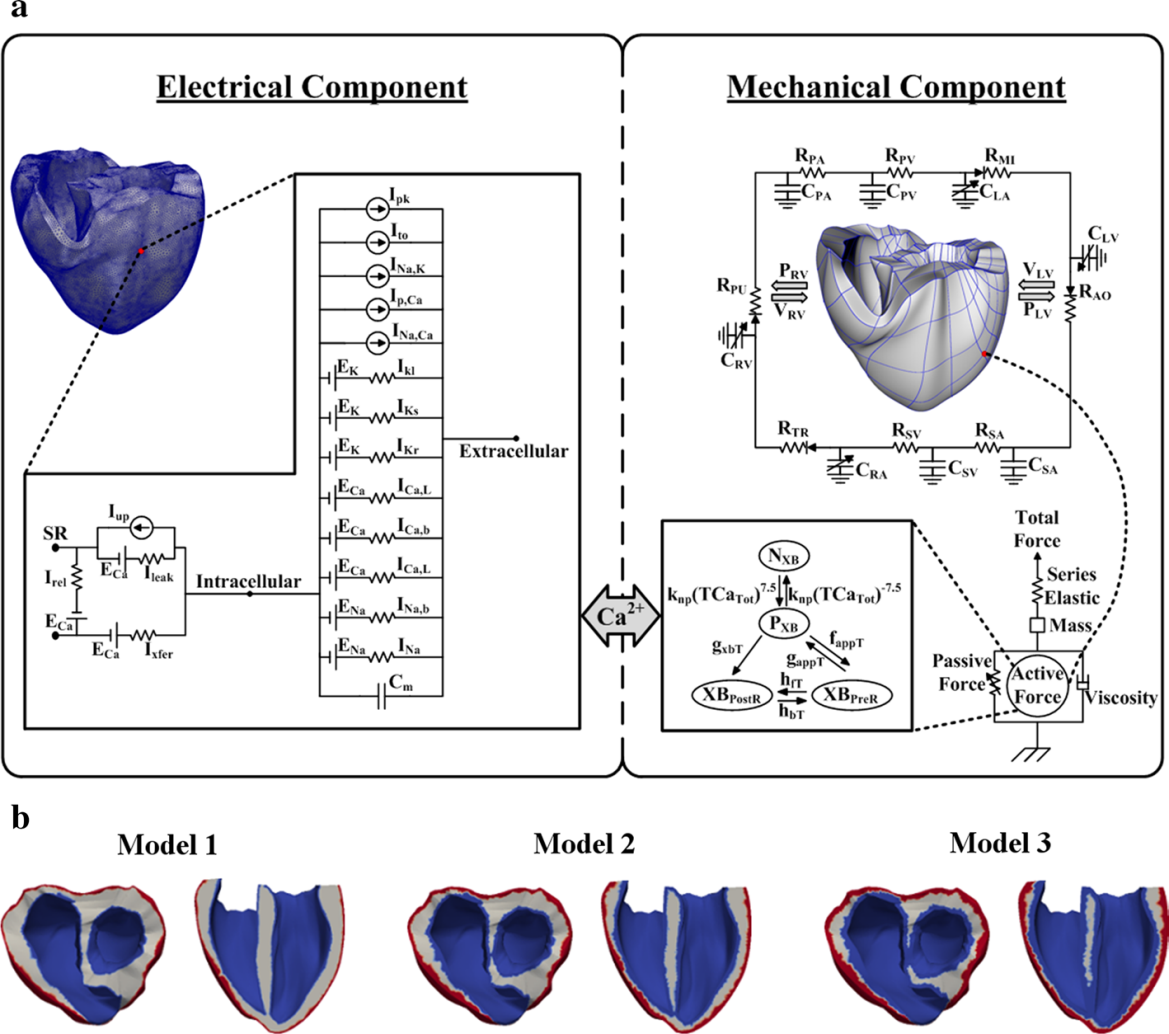
3D models of ventricular tissue. **a** Schematic diagram of the electrical and mechanical components coupled by the Ca^2+^ transient. The electrical component represents the ionic currents and pumps based on the ionic model of Tusscher et al. [36]. The following are shown: the fast inward Na^+^ current (I_Na_), background Na^+^ current (I_Na,b_), L-type inward Ca^2+^ current (I_Ca,L_), background Ca^2+^ current (I_Ca,b_), rapid delayed rectifier K^+^ current (I_Kr_), slow delayed rectifier K^+^ current (I_Ks_), inward rectifier K1 current (I_K1_), Na^+^–Ca^2+^ exchanger current (I_Na,Ca_), sarcoplasmic Ca^2+^ pump current (I_p,Ca_), Na^+^–K^+^ exchange current (I_Na,K_), transient outward K^+^ current (I_to_), K^+^ pump current (I_p,K_), Ca^2+^ release current from the JSR (I_rel_), Ca^2+^ leak current from the JSR (I_rel_), and Ca^2+^ uptake current into the NSR (I_up_). The mechanical component represents the electromechanical model [41] coupled with the circulatory system [28]. The following are shown: the RV pressure (P_RV_), RV volume (V_RV_), LV pressure (P_LV_), LV volume (V_LV_), pulmonary artery resistance (R_PA_), pulmonary artery compliance (R_PV_), pulmonary vein resistance (R_PV_), pulmonary vein compliance (C_PV_), mitral valve resistance (R_MI_), left atrium compliance (C_LA_), systemic vein resistance (R_SV_), systemic vein compliance (C_SV_), tricuspid valve resistance (R_TR_), right atrium compliance (C_RA_), pulmonary valve resistance (R_PU_), non-permissive and permissive conformations of regulatory proteins (N_XB_ and P_XB_, respectively), pre-rotated state bounding with the head extended (XB_PreR_), and post-rotated state illustrating the isomerization for strain inducing the neck region (XB_PostR_). **b** Three heterogeneous models with different thicknesses of the heart-wall layers (epicardium, mid-myocardium, and endocardium)

The mathematical model used in this study consisted of four components: (1) an electrical conduction model of the ventricular tissue, (2) the Purkinje fiber that transmits electrical impulses to the ventricular tissue, (3) a mechanical contraction model of the ventricles, and (4) a lumped-parameter model of the cardiovascular system [28]. Figure 1a shows a schematic of the complete electromechanical model of the electrical and mechanical components and their coupling.

To construct a computational model of the cardiovascular system, we used a 3D finite-element model of a failing human ventricle that was developed in previous studies [27, 29–31] in combination with a lumped-parameter model of the circulatory system [28]. The model was constructed with a ventricular geometry based on MR and DTMR imaging of the heart [27]. According to the imaging data, the ventricles were segmented as explained in previous studies, and a finite-element mesh was generated using the segmented images [32, 33]. Fiber and sheet structural information was also assigned into the mesh. Subsequently, two-dimensional Purkinje fibers, as described by Berenfeld and Jalife [34], were mapped onto the 3D surface of the endocardium in the ventricles [35].

### Electrical model

For the electrophysiological simulation of the ventricles, we used the HyperMesh software to generate finite tetrahedral linear elements (214,319 nodes and 1,061,379 elements). The mesh represents a realistic heart that consists of the epicardium, mid-myocardium, endocardium, and Purkinje fibers. The electrical component of the model simulated the propagation of the AP in the ventricular tissue by solving an electrical conduction equation. This equation represents a continuum of the current flow through cardiomyocytes that are electrically connected. We used the membrane dynamic model proposed by Tusscher et al. [36], which had been validated. The following partial differential equations for reaction–diffusion, which were proposed by Vigmond et al. were used for the electrical conduction in the 3D ventricular tissue [18]:

1 $$\nabla \,.\,\widetilde{\sigma }\nabla V_{m} = \beta I_{m}$$

2 $$I_{m} = C_{m} \frac{{\partial V_{m} }}{\partial t} + I_{ion} (V_{m} ,\upsilon ) - I_{trans} ,$$

where $\tilde{\sigma }\,$ represents the intracellular conductivity, *β* represents the surface-to-volume ratio of the cardiac cells, *C*_*m*_ represents the capacitance of the cells per unit of surface area, *V*_*m*_ represents the membrane potential, and *I*_*trans*_ represents the current density of the transmembrane stimulus. Additionally, *I*_*ion*_ represents the total transmembrane ionic current and is given as [36].

3 $$\begin{aligned} I_{ion} & = I_{Na} + I_{K1} + I_{to} + I_{Kr} + I_{Ks} + I_{CaL} \hfill \\ & \quad + I_{NaCa} + I_{NaK} + I_{pCa} + I_{pK} + I_{bCa} + I_{bNa} , \hfill \\ \end{aligned}$$

where *I*_*Na*_, *I*_*K1*_, *I*_*to*_, *I*_*Kr*_, *I*_*Ks*_, *I*_*Ca,L*_, *I*_*NaCa*_, *I*_*NaK*_, *I*_*pCa*_, *I*_*pK*_, *I*_*bCa*_, and *I*_*bNa*_ represent the rapid inward Na^+^ current, inward rectifier K^+^ current, transient outward K^+^ current, rapid delayed rectifier K^+^ current, slow delayed rectifier K^+^ current, L-type Ca^2+^ current, Na^+^/Ca^2+^ exchanger current, Na^+^/K^+^ pump current, plateau Ca^2+^ current, plateau K^+^ current, background Ca^2+^ current, and background Na^+^ current, respectively.

To model the excitation–contraction (EC) coupling condition, the electrical component was coupled with a mechanical component, as shown in Fig. 1a. Cardiac EC coupling occurs during cellular depolarization in the electrical component, initiating the release of Ca^2+^ from the sarcoplasmic reticulum [37]. Subsequently, the cooperative bindings of Ca to troponin C and cross-bridge cycling occur. The cross-bridge cycling forms the basis for protein movement contractility and the development of active cellular tension, thereby resulting in the deformation of the ventricles. The Ca^2+^ transient response, obtained by the electrical component, serves as an input to the contractile myofilament dynamic model in the mechanical component (see Fig. 1a).

### Mechanical model

The mathematical model of the mechanical contraction of cardiac tissue was based on continuum mechanics [38, 39], with the assumption that the myocardium is hyperelastic and almost incompressible. Additionally, the passive mechanical properties are described as follows [40]:

4 $$W = \frac{C}{2}\left( {e^{Q} - 1} \right)$$

5 $$Q = b_{1} E_{ff}^{2} + b_{2} \left( {E_{rr}^{2} + E_{cc}^{2} + E_{rc}^{2} } \right) + 2b_{3} \left( {E_{fr}^{2} + E_{fc}^{2} } \right),$$

where *W* represents the strain energy function, and E_ij_, the Green–Lagrange strain, represents the local fiber coordinate system. Information on the fiber orientation and laminar sheet was used to determine the orthotropic electrical conductivity and passive mechanical properties of the ventricular myocardium.

In this study, the differential equations of the cross-bridge model of muscle contraction were based on Rice et al. [41]. By assuming an isosarcometric simulation, *dSL/dt* was set as 0, and *SL* (the sarcomere length) was fixed at the initial value *SL*_0_. When the sarcomere contracts, *SL* is computed as follows:

6 $$\frac{d}{dt}SL = \frac{{Integral_{Force} + (SL_{0} - SL) \times viscosity}}{mass},$$

where the viscosity and mass (especially in the mechanical component) are shown in Fig. 1a. *Integral*_*Force*_ represents the normalized forces and is calculated as

7 $$Integral_{Force} = \int\limits_{0}^{1} {\left( {F_{active} (x) + F_{passive} (x) - F_{preload} - F_{afterload} (x)} \right)dt} ,$$

where *F*_*active*_ (*x*) is an active force, *F*_*passive*_ (*x*) is a passive force, and *F*_*preload*_ is a constant force (an applied force that induces an initial *SL*). In the case of *F*_*afterload*_, two conditions can be used: an isotonic contraction or a fixed-muscle length (isometric) contraction. For the isotonic contraction, the afterload force is fixed after the release. Conversely, for the fixed-muscle length contraction, the afterload is calculated as a series elastic element (as shown in the mechanical component in Fig. 1a), as follows:

8 $$F_{afterload} (x) = KSE \times (x - SL_{0} ),$$

where *x* represents the *SL*, and *KSE* represents the stiffness (*Force*/µm).

To simulate the hemodynamic responses, the electromechanical model was coupled with the lumped-parameter model of the circulatory system proposed by Kerckhoffs et al. [28]. This lumped-parameter model is shown in the mechanical component in Fig. 1a and is expressed as follows:

9 $$- R_{SA} \dot{Q}_{SA} + \frac{1}{{c_{SA} }}Q_{SA} = V_{SV}$$

10 $$- R_{SV} \dot{Q}_{SV} + \frac{1}{{c_{SV} }}Q_{SV} = V_{RA}$$

11 $$- R_{RA} \dot{Q}_{RA} + \frac{1}{{c_{RA} }}Q_{RA} = V_{RV}$$

12 $$- R_{RV} \dot{Q}_{RV} + \frac{1}{{c_{RV} }}Q_{RV} = V_{PA}$$

13 $$- R_{PA} \dot{Q}_{PA} + \frac{1}{{c_{PA} }}Q_{PA} = V_{PV}$$

14 $$- R_{PV} \dot{Q}_{PV} + \frac{1}{{c_{PV} }}Q_{PV} = V_{LA}$$

15 $$- R_{LA} \dot{Q}_{LA} + \frac{1}{{c_{LA} }}Q_{LA} = V_{LV}$$

16 $$- R_{LV} \dot{Q}_{LV} + \frac{1}{{c_{LV} }}Q_{LV} = V_{SA} ,$$

where *R* represents the resistance, *Q* represents the flux, *C* represents the compliance, *V* represents the volume, *SA* represents the systemic artery, *SV* represents the systemic vein, *RA* represents the right atrium, *RV* represents the right ventricle, *PA* represents the pulmonary artery, *PV* represents the pulmonary vein, *LA* represents the left atrium, and *LV* represents the left ventricle.

### Heterogeneous myocardium

The heart wall consists of the epicardium (outermost heart layer), mid-myocardium (cardiac muscle tissue in the middle layer of the ventricles), and endocardium (innermost ventricular surface) [2, 3]. In the present study, we used three different 3D ventricular models—called Models 1, 2, and 3—as shown in Fig. 1b. The models differ with regard to the thicknesses of each ventricular layer. Model 1 exhibits the thickest mid-myocardium and the thinnest epicardium and endocardium. Model 3 exhibits the thinnest M cell layer and the thickest epicardium and endocardium. Model 2 exhibits intermediate-thickness layers of the heart walls.

At a position 1 cm from the base (see Fig. 1b), the proportions of the epicardium and endocardium layers of Model 1 were 12% in the right ventricle (RV) wall and 9% in the left ventricle (LV) wall. The total percentage of the endocardium layer was 22% in the septum wall (11% in each side of the septum wall—right and left). In Model 2, the proportions of the epicardium and endocardium layers were 23.5% in the RV wall and 18% in the LV wall. The endocardium layer in Model 2 corresponded to 19% for each side of the septum wall. In Model 3, the proportions of the epicardium and endocardium layers were 41% in the RV wall, 22% in the LV wall, and 26% for each side of the septum wall.

### Simulation protocol

We investigated two scenarios: (1) a normal sinus rhythm, representing the control group, and (2) reentry, mimicking a pathological condition. We observed and compared the electromechanical responses of three different ventricular models (Models 1, 2, and 3) during the normal sinus rhythm and reentry, which was associated with a tachyarrhythmia condition. For each scenario, we performed two simulations: electrical and mechanical. For the normal sinus rhythm, a basic cycle length (BCL) of 600 ms was used for the electrical simulation. The sinus rhythm was performed for 3 s to obtain a steady-state condition. However, only the data for the last cycle (2.4 to 3 s) were used to simulate the ventricular electromechanics. The electrical impulse was initiated via Purkinje fibers with an electrical conduction velocity (CV) of 200 cm/s. Additionally, a myocardial CV (MCV) of 70 cm/s was used for the normal condition [6].

For the electrical simulation with the normal sinus rhythm, the electrical activation time (EAT) and electrical deactivation time (EDT) were calculated. The EAT and EDT are the times required for tissue depolarization and cellular repolarization, respectively. In the simulation, the EAT was the time when the membrane potential of the ventricles exceeded − 30 mV, and the EDT was the time when the membrane potential of the ventricles decreased below − 75 mV.

The reentry scenario was initiated using an S1–S2 protocol that was introduced by Tusscher et al. [36]. Three S1 stimuli were applied in the apex of the ventricles with a BCL of 600 ms. The stimuli generated waves that propagated in all directions. After the refractory tail of the wave passed through half of the length of the medium, an S2 stimulus was applied parallel to the S1 stimulus at three-quarters of the medium length. This produced a second wavefront with a curly tip and generated a reentrant spiral wave.

In the present study, the ventricular electromechanical simulations of the reentry scenario were conducted over 10 s, and two different initialization methods were used to examine the reentry arrhythmogenesis and the instability of the reentrant waves in the electrical simulation. In the first initialization method, the electrical simulation was initiated via the S1–S2 protocol as described previously, and the MCV was 43 cm/s. In the second method, the initial condition was from the outputs that were derived from the previous electrical simulation using the S1–S2 protocol that already generated reentry. The MCV of this method was 70 cm/s.

The results of the electrical simulation for both the sinus rhythm and the reentry conditions were coupled with the intracellular Ca^2+^ transient and were used as inputs for the mechanical simulation. Subsequently, the results of the mechanical simulation were used to analyze the ventricular mechanical responses, including the pressure, volume, total adenosine triphosphate (ATP) consumption rate, ejection fraction (EF), stroke volume (SV), stroke work (SW), and cardiac output (CO).

The SV represents the amount of blood pumped from the LV and is obtained by subtracting the end systolic volume (ESV) from the end diastolic volume (EDV). The EF represents the amount of blood ejected from the ventricles with each heartbeat and is obtained by dividing the SV by the EDV and multiplying the result by 100%. The SW corresponds to the work or pressure of blood processed in one cycle during ventricular contraction and is obtained by integrating the enclosed area of the pressure–volume (P–V) curve (Fig. 4b). The CO represents the amount of blood pushed out by the heart per minute.

## Results

### Ventricular electromechanical responses during normal sinus rhythm

**Fig. 2:**
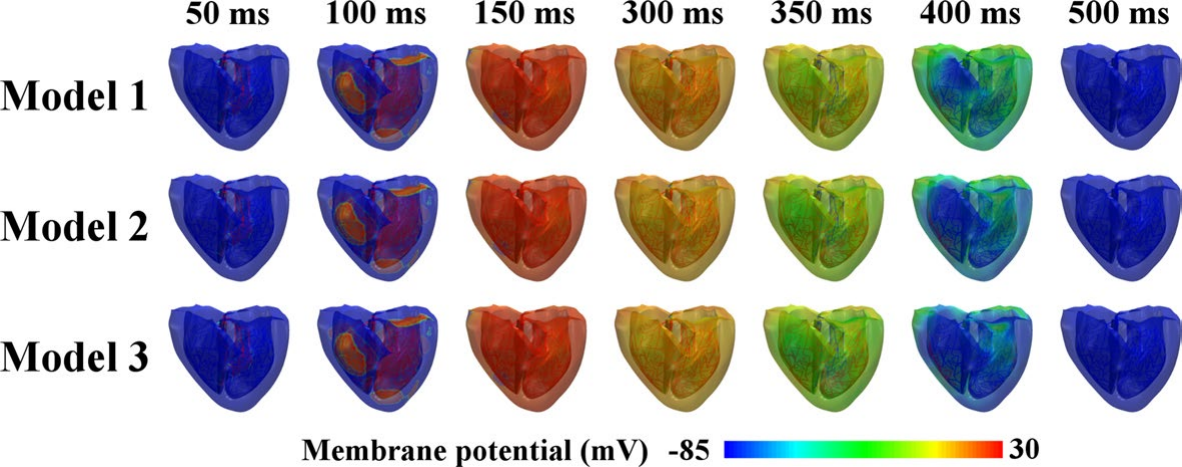
Membrane potential propagation of sinus pacing in time-series for the three models

Figure 2 shows the transmural distribution of the membrane potentials of the three different ventricular models during a normal sinus rhythm. The membrane potential was scaled between − 85 and 30 mV, as shown by the color scale from blue to red. The three cardiac models repolarized differently despite depolarizing in the same pattern. Model 1 (which exhibited the thickest M cell layer) experienced the slowest repolarization phase, and Model 3 (which exhibited the thinnest mid-myocardium) repolarized faster than Model 2. This is clearly observed in the illustrations for 400 ms (see Fig. 2), where Model 1 exhibited larger green regions, indicating late repolarization. Conversely, early repolarization was observed in Model 3, which showed larger blue regions at 400 ms. However, all the ventricular regions of all three models were completely repolarized in the last snapshot, i.e., 500 ms.

**Fig. 3:**
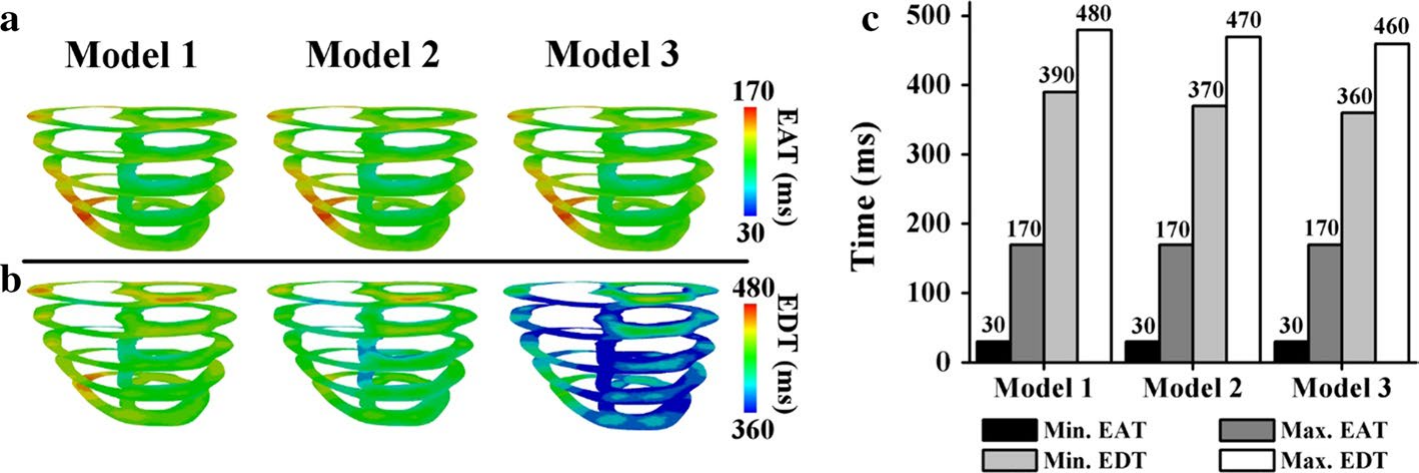
Electrical activation time (EAT) and electrical deactivation time (EDT) during the last cycle of a sinus rhythm. **a** EAT. **b** EDT. **c** Comparisons of the minimum and maximum EAT and EDT

Figure 3 compares the EAT and EDT of ventricles during the sinus rhythm in the last cycle. The minimum EAT represents the earliest local depolarization time in the ventricles (i.e., the Q wave in an electrocardiogram). Conversely, the maximum EAT represents the time at which all the tissues depolarized (i.e., the S wave in an electrocardiogram). The minimum EDT represents the time at which the tissues started to repolarize (i.e., the beginning of the T wave in an electrocardiogram), and the maximum EDT represents the time at which all the tissues entirely repolarized (i.e., the end of the T wave in an electrocardiogram).

As clearly shown in Fig. 3, the three different ventricular models exhibited similar EAT patterns (Fig. 3a), with a maximum EAT of 170 ms, as indicated in red. The minimum EAT is represented by dark blue, with an average of 30 ms, because the Purkinje terminal node activated the ventricular tissue at approximately 30 ms. However, the EDT maps of the three models differ significantly (Fig. 3b). The EDT scale ranges from 360 to 480 ms, as denoted by the color scale from blue to red. As indicated by the green ventricles in Fig. 3b, Model 1 repolarized more slowly than the other two models. Conversely, the colors of Model 3 were mainly blue, indicating faster repolarization than Model 2.

A closer examination of the bar chart in Fig. 3c indicates that the minimum and maximum EATs of the three models were 30 and 170 ms, respectively. Regarding the minimum EDT, Models 1, 2, and 3 exhibited different repolarization initiation times: 390, 370, and 360 ms, respectively. Model 1 exhibited the longest maximum EDT of 480 ms, and Model 3 exhibited the shortest EDT of 460 ms.

**Fig. 4:**
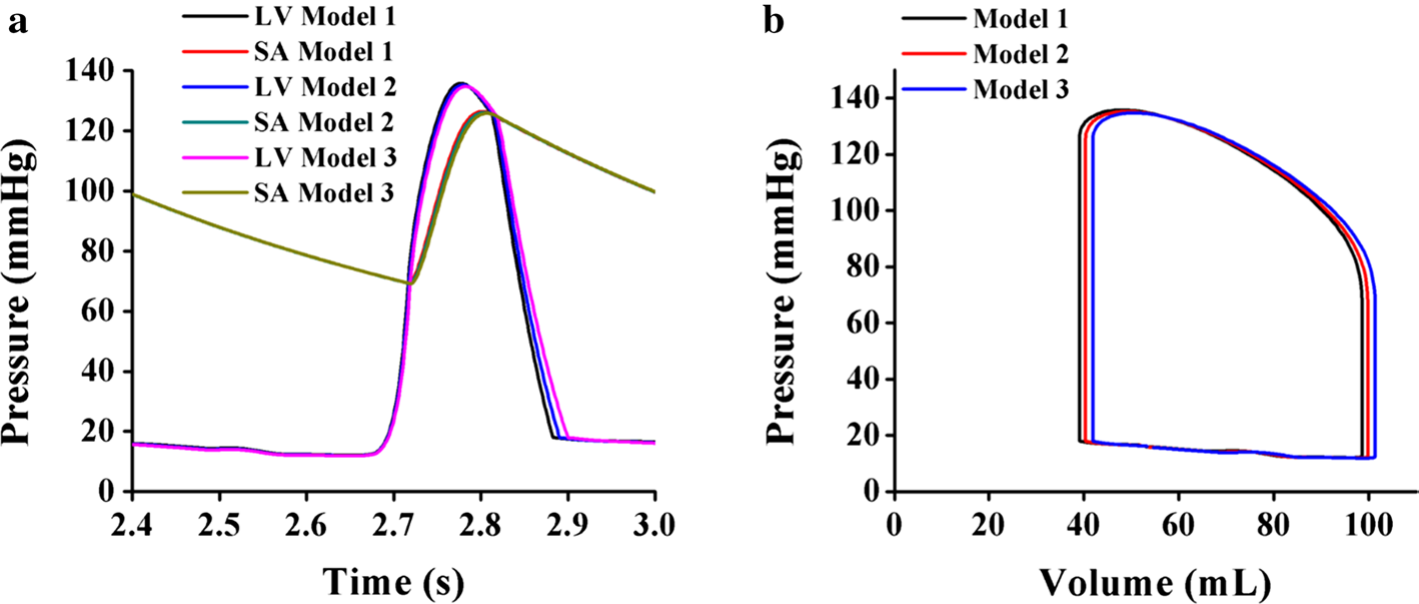
Cardiac mechanical responses during the last cycle of a sinus rhythm. **a** Pressure graph for the LV and SA. **b** Pressure–volume (P–V) graph for the LV

Figure 4 illustrates the pressure and the relationship between the pressure and volume (P–V) responses of the LV in the last cycle during the sinus rhythm for Models 1, 2, and 3. Figure 4a compares the pressures of the LV and the systemic artery (SA) for Models 1, 2, and 3. Although the SA and LV peak pressures did not significantly differ among the models, Model 1 generated the highest LV peak pressure at approximately 136 mmHg, and its systolic and diastolic aortic pressures were 126 and 69 mmHg, respectively.

As shown in Fig. 4b, Model 1 exhibited the lowest ESV and EDV at approximately 39 and 97 mL, respectively. In comparison, for Models 2 and 3, the volume shifted to the right, and the ESV and EDV increased by 5% and 2%, respectively. Interestingly, all the models exhibited similar patterns for both the end systolic pressure and the end diastolic pressure of the LV.

Table 1 shows a comparison of the ventricular mechanical responses for Models 1, 2, and 3 in the last cycle during the normal sinus rhythm, including the SV, EF, SW, CO, and total ATP consumption rate. The results indicate that Model 1 generated the highest values of the numerical data, followed by Models 2 and 3.

**Table 1:** Ventricular mechanical responses during a normal sinus rhythm, including the SV, EF, SW, CO, and total ATP consumption rate for the three models

Model	SV (mL)	EF (%)	SW (mmHg mL)	CO (L/min)	Total ATP consumption rate (s^−1^)
Model 1	59	60	6293	5.95	65
Model 2	59	59	6265	5.94	61
Model 3	59	58	6238	5.93	58

All three models pumped the same blood volume (SV), i.e., approximately 59 mL. The largest EF was exhibited by Model 1 (60%), and the EFs for Models 2 and 3 were 59% and 58%, respectively. Additionally, Model 1 exhibited the highest SW (6293 mmHg mL), followed by Model 2 (6265 mmHg mL) and Model 3 (6238 mmHg mL). The highest CO was exhibited by Model 1 (5.95 mL). The consumption of contractile ATP for Models 1, 2, and 3 was 65, 61, and 58 s^−1^, respectively.

### Ventricular electromechanical responses during reentry

**Fig. 5:**
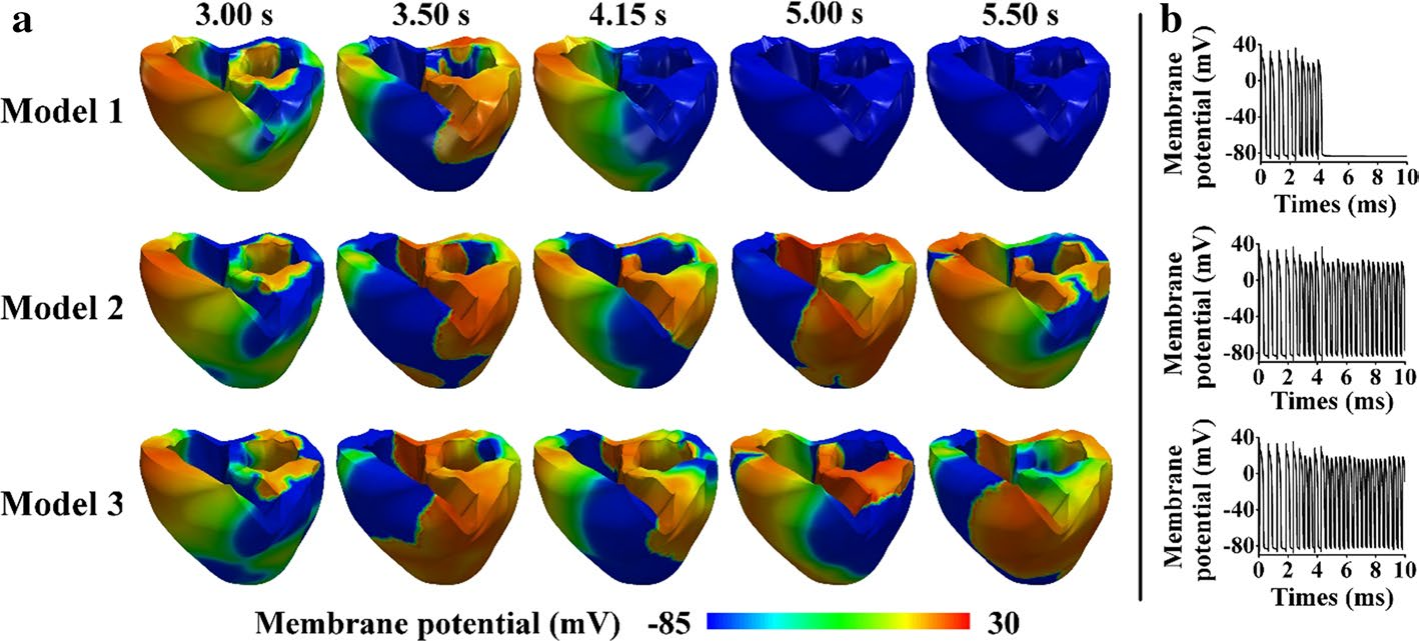
Ventricular electrical responses during reentry, in which the initial condition uses the S1–S2 protocol (MCV of 43 cm/s). **a** Reentrant spiral waves in the 3D ventricular tissue models. **b** Membrane potentials of the ventricles

Figure 5 shows a comparison of the cardiac electrical responses [including snapshots of the reentrant spiral waves (Fig. 5a) and the membrane potentials of the ventricles (Fig. 5b)] during reentry, where the simulation was initiated via the S1–S2 protocol (MCV of 43 cm/s), as described in “Methods” section. As shown in Fig. 5a, Model 1 began to experience reentry termination at 4.15 s and exhibited complete termination at 5 s, as indicated by the ventricles that were entirely blue. Conversely, for Models 2 and 3, the reentry was sustained, as indicated by the reentrant spiral waves propagating until the end of the simulation (Fig. 5a). Additionally, when the ventricles induced reentry and generated reentrant spiral waves, the ventricles produced membrane potentials, as shown in Fig. 5b. The reentry for Model 1 terminated at approximately 4 s, whereas for Models 2 and 3, the reentry was sustained until the end of the simulation (for 10 s).

**Fig. 6:**
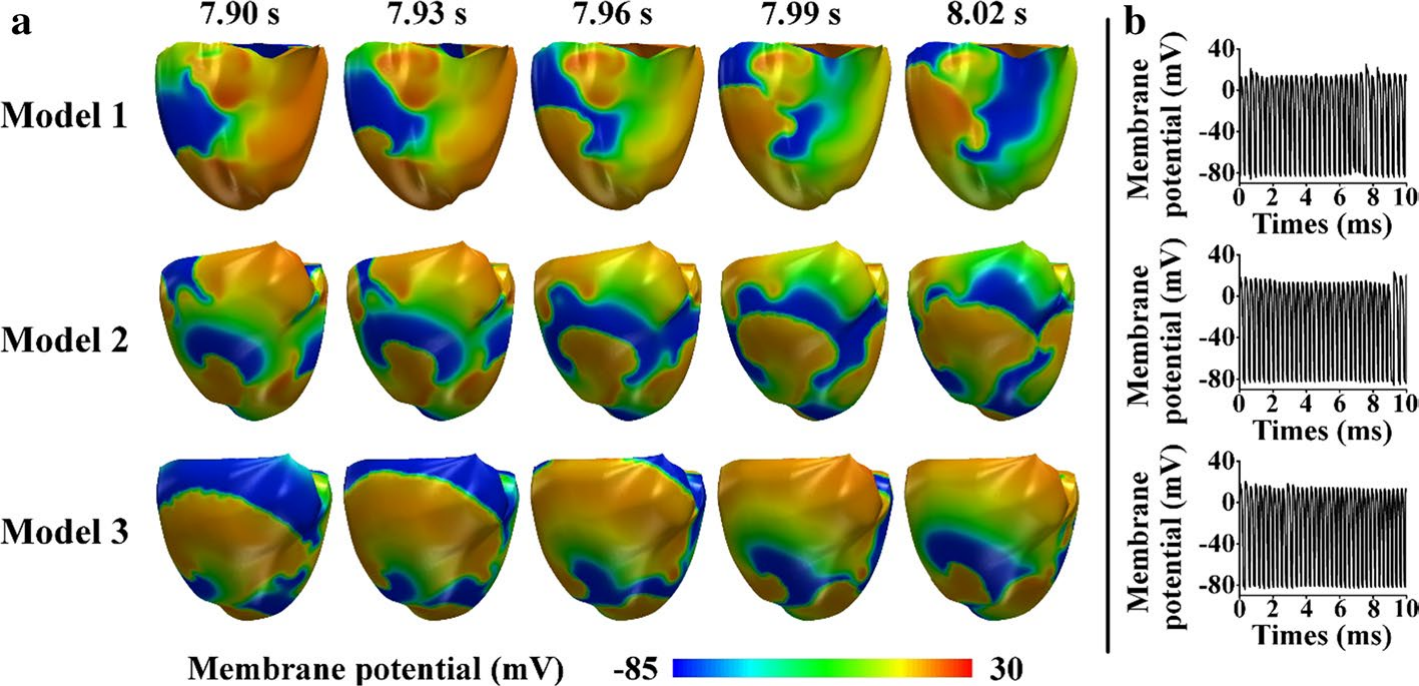
Cardiac electrical responses during reentry, in which the initial condition was based on the outputs derived from the previous electrical simulation using the S1–S2 protocol that already generated reentry (MCV of 70 cm/s). **a** Reentrant spiral waves in the 3D ventricular tissue models. **b** Membrane potentials of the ventricles

Figure 6 illustrates the ventricular electrical responses, which indicate the instability of the spiral waves during reentry, with an MCV of 70 cm/s for the three models. The initial conditions of this scenario corresponded to the outputs that were derived from the previous electrical simulation using the S1–S2 protocol that already generated reentry. Snapshots of the spiral waves of the reentry are shown in Fig. 6a, and b shows the membrane potentials of the ventricles. For all three models, reentry was sustained until the end of the simulation, as indicated by the generation of reentrant spiral waves until the end of the simulation (Fig. 6a). Some rotation centers of reentrant spiral waves were located at different regions of the ventricles simultaneously. Therefore, the rotors collided and generated spiral-wave breakups. Additionally, the membrane potentials were generated in all three models over the simulation period of 10 s (Fig. 6b).

Table 2 presents the total number of rotors at several times during reentry, where the initial condition employed the outputs, which were derived from the previous electrical simulation using the S1–S2 protocol that already generated reentry. All models generated the highest number of rotors at 8 s. Model 1 exhibited the smallest number of rotors (five), and Model 3 exhibited the largest number of center rotations (eight). Model 2 exhibited seven rotors at 8 s.

**Table 2:** Total number of rotors during reentry where the initial condition is from the outputs derived from the previous electrical simulation using the S1–S2 protocol that already generated reentry (MCV is 70 cm/s)

Model	Number of rotors			
	4 s	6 s	8 s	10 s
Model 1	4	3	5	2
Model 2	5	4	7	3
Model 3	3	5	8	5

**Fig. 7:**
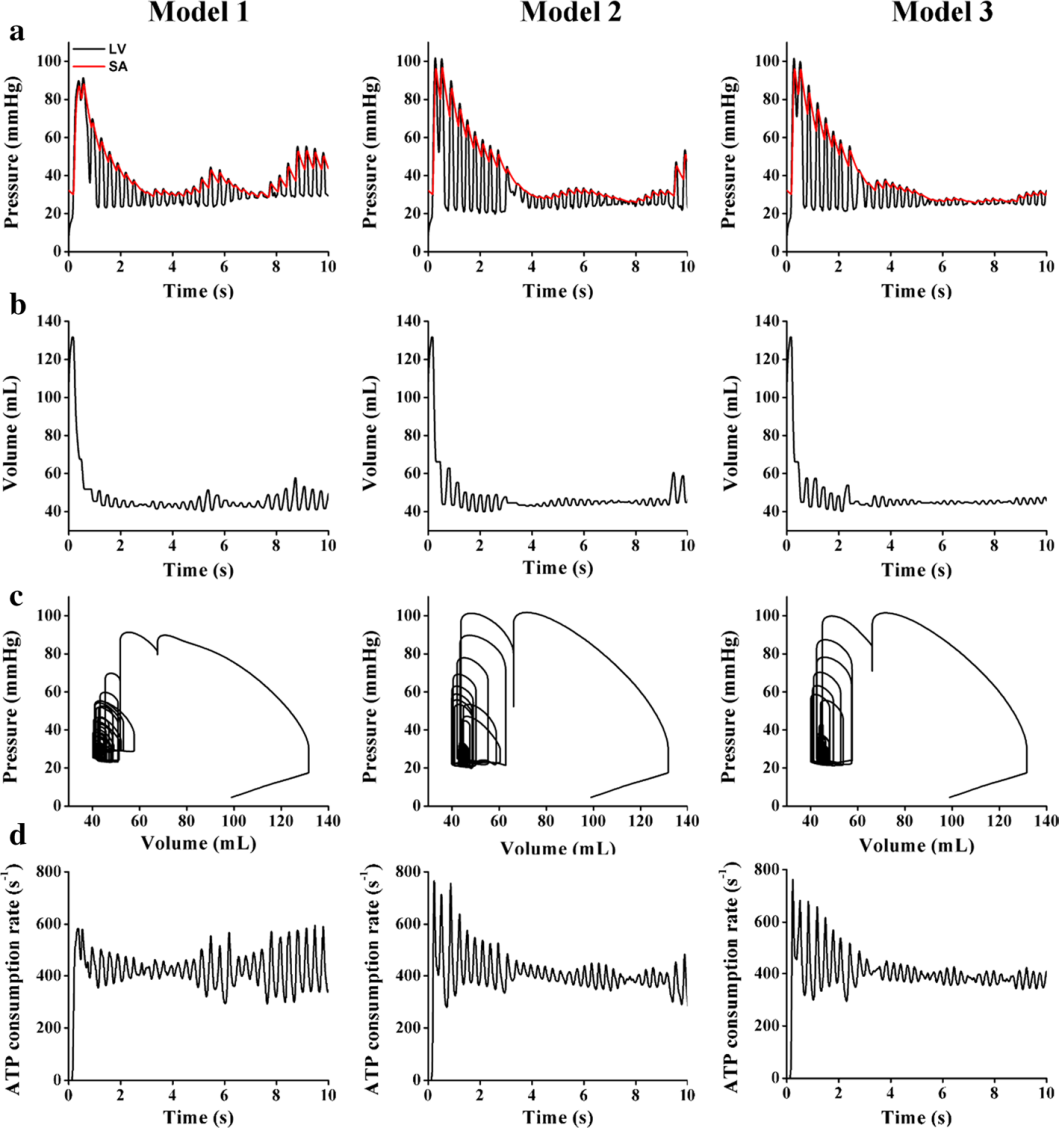
Ventricular mechanical responses during reentry, in which the initial condition was based on the outputs derived from the previous electrical simulation using the S1–S2 protocol that already generated reentry (MCV of 70 cm/s). **a** LV and SA pressure graphs. **b** Volume charts. **c** P–V curves. **d** ATP consumption-rate graphs

Figure 7 compares the ventricular mechanical responses of the three models during reentry, in which the initial condition employed the outputs, derived from the previous electrical simulation using the S1–S2 protocol that already generated reentry. The results include the LV and SA pressure charts (Fig. 7a), LV volume graphs (Fig. 7b), pressure–volume relation curves (Fig. 7c), and ATP consumption rates of the ventricles (Fig. 7d). The data in Fig. 7a–c fluctuated during reentry throughout the simulation period of 0–10 s. Transient responses occurred at the beginning of the simulation time between 0 and 4 s, which was attributed to the initial state in our computational model.

As shown in Fig. 7a, the LV pressure slightly exceeded the SA pressure over the entire period. Despite similar fluctuations of both pressure waveforms for the three models, Model 1 exhibited the most erratic patterns for both the LV and SA pressures. In Model 1, after the transient responses (0–4 s), both pressures gradually increased from approximately 30 mmHg (at 4 s) to approximately 40 mmHg (at 6 s). Although the pressures significantly declined to 25 mmHg over the next 2 s, they increased sharply to 53 mmHg at the end of the simulation. In Model 2, although there were steady oscillations of the pressures between 4 and 9.5 s at approximately 28 mmHg, the pressures rapidly increased by 45% in the last 10 s. In Model 3, the LV and SA pressures leveled off at approximately 24 mmHg for the entire simulation period after the transient time.

Figure 7b shows a comparison of the LV volume for the three models. Interestingly, the pattern of the volume waveforms over the period is similar to that of the pressure graphs (Fig. 7a). Although the volumes of the three models fluctuated, the largest LV volume was generated by Model 1. In Model 1, after the transient time (4 s), the volume steadily increased from 42 to 51 mL at 5 s. Subsequently, although the model exhibited an LV volume reduction of 18% over the next 2 s, the volume increased rapidly to approximately 53 mL at the end of the simulation. In Model 2, the LV volume remained constant at 47 mL between 4 and 9 s and then suddenly increased to approximately 58 mL at the end of the simulation. Model 3 pumped the smallest amount of blood (approximately 45 mL) in the simulation period.

The relationship between the pressures (Fig. 7a) and volumes (Fig. 7b) is described by the P–V curves shown in Fig. 7c. As shown in Figs. 6b and 7a, the pressures and volumes fluctuated over the simulation period (10 s); thus, the P–V curves vacillate for all three models.

Figure 7d shows the total ATP consumption rate during reentry. In Model 1, it exhibited the highest ATP consumption rate, and the total ATP consumption rate increased gradually after the transient time (instead of fluctuating), reaching a maximum value of almost 600 s^−1^ at 10 s. Model 3 exhibited the lowest ATP consumption rate (approximately 400 s^−1^), and Model 2 exhibited a slightly higher rate (average of 440 s^−1^).

**Fig. 8:**
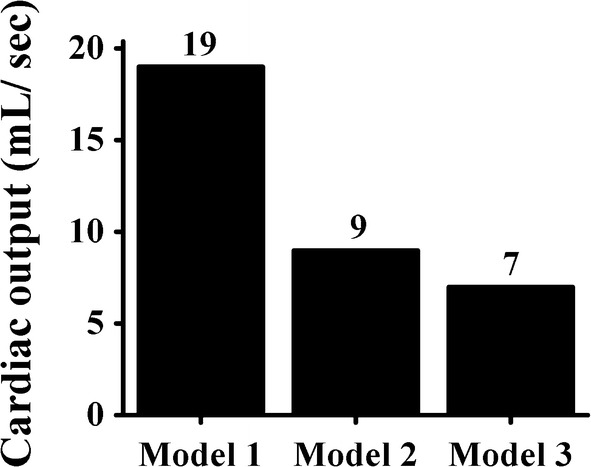
Cardiac output (CO) graphs of mechanical simulations in the reentrant condition, where the initial condition was based on the outputs derived from the previous electrical simulation using the S1–S2 protocol that already generated reentry (MCV of 70 cm/s)

Figure 8 shows the CO, i.e., the amount of blood ejected from the ventricles owing to the cardiac mechanical responses during reentry, where the initial condition was based on the outputs, derived from the previous electrical simulation using the S1–S2 protocol that already generated reentry. Model 1 exhibited the highest CO (19 mL/s), and the CO of Model 2 was slightly less than 50% of that of Model 1. Model 3 exhibited a CO of only 7 mL/s.

## Discussion

The objective of this study was to observe the effects of three electromechanical ventricular models with different thicknesses of the heart-wall layers (epicardium, mid-myocardium, and endocardium), as each heart wall exhibits different characteristics and possible variations in the layer size. The three models were denoted as Models 1, 2, and 3. As explained previously, Model 1 exhibited the thickest mid-myocardium and the thinnest epicardium and endocardium; Model 3 exhibited the thinnest M cell layer and the thickest epicardium and endocardium; and Model 2 exhibited intermediate thicknesses of the epicardium, mid-myocardium, and endocardium. We compared the ventricular electromechanical responses (in the cases of a normal sinus rhythm and reentry) of the three different models. The main findings are as follows.

1. The ventricles generated longer wavelengths when the thickness of the mid-myocardium increased. Therefore, the ventricles required a longer time for repolarization, yielding a longer EDT.
2. In the case of cardiac arrhythmogenesis simulated using the S1–S2 protocol with an MCV of 43 cm/s, the ventricles generated a terminated reentry and reduced arrhythmogenicity in the presence of a thick mid-myocardium. More sustainable reentrant waves were generated when the ventricles had thinner M cell layers.
3. A thinner mid-myocardium caused the ventricles to generate more rotors, experience more severe spiral-wave breakups, and have a lower pumping strength.
4. The heterogeneity effects of the heart-wall layer thicknesses were concealed in the normal sinus rhythm condition. However, the heterogeneity significantly affected the ventricular responses in the reentry scenario, where the pressure, volume, and total ATP consumption rate (resulting from mechanical responses during reentry) were significantly higher than those under the normal sinus rhythm condition.
5. With the decrease of the thickness of the mid-myocardium, the performance of the ventricles worsened with regard to contractility and pumping efficacy.

To the knowledge of the authors, the wavelength of the electrical impulse in the mid-myocardium (in which the cell exhibits the longest APD among the three cell types) is the longest among the three layers at the same electrical CV. This is because the wavelength of the electrical impulse is proportional to the APD and CV. Model 1 exhibits the thickest M cell layer; thus, it generates the longest wavelength. Hence, Model 1 requires the longest time to repolarize entire ventricles, as indicated by the longest minimum and maximum EDTs (390 and 480 ms, respectively) (Fig. 3c). Conversely, Model 3 exhibits the thinnest mid-myocardium; thus, it generates a shorter wavelength than Models 1 and 2. Therefore, Model 3 exhibits the fastest repolarization and the shortest minimum and maximum EDT values (360 and 460 ms, respectively) (Fig. 3c).

As shown in Fig. 5a, the reentrant wave terminates when the ventricles exhibit thicker M cell layers. Conversely, the ventricles generate a more sustainable reentry in the presence of thinner mid-myocardial layers. Model 1 begins to terminate at approximately 4 s, as indicated by the ventricles that turn completely blue. The thicker M cell layer contains larger areas that produce longer excitation wavelengths; thus, there are more limited resting areas to propagate. Hence, the reentrant wave exhibits a greater probability of termination, and the ventricles return to their normal condition, as shown in Model 1, which has the thickest M cell layer.

For all three models, the largest number of rotors over the 10-s period occurs at 8 s (Table 2). At this time, Model 1 exhibits the lowest number of center rotations (five rotors), and Models 2 and 3 exhibit seven and eight rotors, respectively. This is because Model 1 exhibits the longest wavelength, as it has the thickest M cell layer. Conversely, Model 3 exhibits the thinnest M cell layer; thus, the wavelength is shorter than those of Models 1 and 2. Because of the shorter wavelength, the ventricles of Model 3 have more opportunities to propagate and break the spiral waves into several rotors. Hence, the rotors exhibit more possibilities to collide, resulting in more chaotic and unstable spiral waves in Model 3. Thus, an increase in the number of rotors causes the spiral waves to become more chaotic and unstable.

To investigate the effects of the heterogeneous layer thicknesses of the ventricles on the ventricular mechanics, we analyzed the hemodynamic mechanical responses of each model under both normal sinus rhythm and reentry conditions. These responses included the pressure, volume, SV, EF, SW, CO, and total ATP consumption rate. The results indicate that the heterogeneity of the heart-wall layer thicknesses is concealed in the normal sinus rhythm scenario (Fig. 4 and Table 1). For example, the gap numbers of the LV and SA peak pressures differ only slightly (Fig. 4a)—by no more than 1 mmHg—among the three models. However, the thickness heterogeneity of the heart-wall layers significantly affects the pathological condition, as indicated by the mechanical responses in the reentry scenario (Fig. 7). For example, we consider the pressures at approximately 6 s (Fig. 7a), and the LV and SA peak pressures of Model 1 tend to be significantly higher at 40 mmHg than those of Model 2 (30 mmHg) and Model 3 (24 mmHg).

The peak LV pressure decreases in the presence of a thinner mid-myocardium in the ventricles (Figs. 4a and 7a). The LV pressure is proportional to the blood volume pumped from the ventricles (Fig. 7b). In the presence of the thinner M cell layer in the ventricles, the volume of ejected blood tends to decrease (Fig. 7b), and the P–V loop is slightly reduced (Fig. 4b). Hence, the CO resulting from the mechanical responses is likely to be significantly higher when the ventricles contain thicker M cell layers (Fig. 8). Reductions in the LV pressure, blood volume, and CO indicate that the ventricles exhibit lower pumping strength throughout the circulation. Therefore, Model 3, which has the thinnest M cell layer, shows the lowest pumping pressure and does not eject enough blood to satisfy the metabolic requirements of the human body, because it pumps less blood than Model 1. Hence, Model 3 exhibits low values of the SV, EF, and SW (Table 1). For all the models, the LV peak pressure slightly exceeds the SA pressure. This implies that the blood is successfully pumped in the circulatory system.

Regarding the total ATP consumption rate (Table 1 and Fig. 7d), the ATP consumption increases if the ventricles have thicker M cell layers. This is because ventricles with longer wavelengths require more energy to contract. Thus, the ventricles produce more ATP. Therefore, Model 1 generates the longest wavelength, consumes the largest amount of energy for ventricular contraction, and exhibits the highest ATP consumption rate.

Interestingly, a strong correlation exists between the total number of rotors (Table 2) and the mechanical responses (Fig. 7). Specifically, the total number of center rotations is not proportional to the mechanical outputs. For example, in the case of the pressure waveforms (Fig. 7a), the pressure tends to increase when the number of central rotations decreases. Conversely, the pressure exhibits a decreasing trend when the ventricles generate more rotors. This implies that increasing the number of rotors reduces the pumping strength of the ventricles. For example, the pressure is low when Model 1 generates four rotors at 4 s. Subsequently, over the next 2 s, the pressure in Model 1 increases while the total number of central rotations decreases. At 8 s, there is an increase in the number of rotors, although the pressure decreases. At the end of the simulation period, the number of rotors declines to two; thus, the pressure is more likely to increase.

The results of this study elucidate the effects of myocardial heterogeneity (i.e., different thickness ratios of the epicardium, mid-myocardium, and endocardium) on the pumping performance. The different thickness ratios can be considered as causes of heart arrhythmia in clinical practice. For instance, in the case of a patient with heart failure, the epicardium, mid-myocardium, and endocardium thickness ratio in the ventricles could be the reason why the heart does not eject blood properly.

Several limitations should be considered. First, we did not use clinical or experimental data in the present study. We conducted the simulation using electromechanical models and methodologies from previous studies [6, 27–31, 41]. For the cardiac electrophysiological simulation, we used the human ventricular model proposed by Tusscher et al. [6], and the myofilament dynamics model described by Rice et al. was applied to the mechanical model [41]. Subsequently, we used the computational model of failing human ventricles. Additionally, a one-way EC coupling model was used to ensure that the cardiac electrical behavior was not affected by the ventricular mechanical activity.

## Conclusions

The results of the study prove that the thicknesses of the heart-wall layers affect the ventricular electromechanical responses in the cases of normal sinus rhythm and reentry. The ventricles that contained thicker M cell layers generated higher mechanical responses, e.g., the pressure, volume, and CO, under both the normal and reentry conditions. Therefore, they had better pumping efficacy and contractility and a lower probability of developing arrhythmia (arrhythmogenesis). Conversely, the ventricles with thinner mid-myocardial layers generated more chaotic and unstable reentrant waves and exhibited worsened ventricular pumping.

## Abbreviations

AP: action potential
APD: action-potential duration
BCL: basic cycle length
CO: cardiac output
CV: conduction velocity
EAT: electrical activation time
EC: excitation–contraction
EDT: electrical deactivation time
EDV: end diastolic volume
EF: ejection fraction
ESV: end systolic volume
LV: left ventricular
MCV: myocardial conduction velocity
P–V: pressure–volume
RV: right ventricular
SA: systemic artery
SV: stroke volume
SW: stroke work
